# Flunitrazepam increases the risk of lamotrigine‐induced cutaneous adverse reactions: Combined analysis of medical big data and clinical research

**DOI:** 10.1111/pcn.13866

**Published:** 2025-07-17

**Authors:** Hiroshi Bando, Mitsuhiro Goda, Masahito Nakataki, Keita Hirai, Yuki Nitta, Kei Kawada, Toshihiko Yoshioka, Masaya Kanda, Atsushi Ogawa, Chiaki Mukuta‐Murakami, Kaito Tsujinaka, Koji Miyata, Kohei Kitagawa, Takahiro Niimura, Fuka Aizawa, Kenta Yagi, Yuki Izawa‐Ishizawa, Masayuki Chuma, Takafumi Naito, Yoshikazu Tasaki, Shusuke Numata, Keisuke Ishizawa

**Affiliations:** ^1^ Department of Clinical Pharmacology and Therapeutics, Graduate School of Biomedical Sciences Tokushima University Tokushima Japan; ^2^ Department of Pharmacy Tokushima University Hospital Tokushima Japan; ^3^ Department of Clinical Pharmacology and Therapeutics, Graduate School of Biomedical and Heath Sciences Hiroshima University Hiroshima Japan; ^4^ Department of Psychiatry, Graduate School of Biomedical Sciences Tokushima University Tokushima Japan; ^5^ Department of Pharmacy Shinshu University Hospital Matsumoto Japan; ^6^ Department of Clinical Pharmacology and Therapeutics Shinshu University Graduate School of Medicine Matsumoto Japan; ^7^ Department of Hospital Pharmacy and Pharmacology Asahikawa Medical University Asahikawa Japan; ^8^ Department of Neuropsychiatry Okayama Psychiatric Medical Center Okayama Japan; ^9^ Clinical Research Center for Developmental Therapeutics Tokushima University Hospital Tokushima Japan; ^10^ Department of Pharmacy Shimane University Hospital Izumo Japan; ^11^ Department of Health and Nutrition, Faculty of Human Life Science Shikoku University Tokushima Japan

**Keywords:** flunitrazepam, lamotrigine, medical big data, rash, UDP‐glucuronosyltransferase

## Abstract

**Aim:**

The concomitant use of lamotrigine and valproic acid, a uridine diphosphate (UDP)‐glucuronosyltransferase inhibitor, is a known risk factor for lamotrigine‐induced rashes, which may develop into fatal or severe cutaneous adverse reactions. No drugs other than valproic acid have been shown to increase lamotrigine concentrations in humans by inhibiting UDP‐glucuronosyltransferase. The treatment of bipolar disorder and epilepsy is often combined with other medications, which may affect the risk of rash. We aimed to identify medications that increased the risk of lamotrigine‐induced rashes using clinical data.

**Methods:**

We used VigiBase to search for drugs associated with the increased number of reported severe cutaneous adverse reactions when combined with lamotrigine. In a retrospective study, we collected information on patients from electronic medical records and used propensity score matching to compare the incidence of lamotrigine‐induced rash with and without flunitrazepam use. Furthermore, we compared lamotrigine concentrations between patients treated with and without flunitrazepam in a prospective observational study.

**Results:**

VigiBase analysis showed that flunitrazepam significantly increased the reporting odds ratio for lamotrigine‐related severe cutaneous adverse reactions. In the retrospective study, combining lamotrigine with flunitrazepam significantly increased the incidence of rashes. In the prospective observational study, lamotrigine with flunitrazepam significantly increased the concentration‐to‐dose ratio.

**Conclusion:**

Flunitrazepam use may affect lamotrigine concentration and increase the risk of rash. These results have implications for the choice of sleep medication. In addition, when lamotrigine is used in combination with flunitrazepam, the lamotrigine dosing design should address lamotrigine‐induced rash management.

Lamotrigine (LTG) is an effective prophylactic agent for bipolar disorder and is used to treat a variety of psychiatric disorders.^1^ In addition, it is a broad‐spectrum antiepileptic drug, the first‐line therapy for focal seizures in children and adults, and has shown efficacy against generalized seizures.^2^ Moreover, LTG is less teratogenic and preferred for girls and women of childbearing potential.^3^ Given these characteristics, LTG plays a crucial role in the treatment of psychiatric and neurological diseases. Despite its therapeutic benefits, LTG is associated with severe cutaneous adverse reactions (SCARs), such as Stevens–Johnson syndrome and toxic epidermal necrolysis, which can be life‐threatening in rare cases.^4^, ^5^

Factors associated with SCARs in patients taking LTG include concomitant use of valproic acid (VPA),^6^ doses exceeding the protocol,^6^ age younger than 13 years,^7^ history of rash from other antiepileptic drugs,^7^ and being within 8 weeks of LTG treatment.^8^ One of these factors, high serum concentrations of LTG in the early stages of administration, is associated with the development of rashes.^6^, ^8^, ^9^, ^10^ LTG is primarily metabolized by uridine diphosphate (UDP)‐glucuronosyltransferase (UGT) to LTG N2‐glucuronide.^11^, ^12^ VPA is a UGT inhibitor, and its concomitant use decreases the total clearance of LTG and increases its elimination half‐life.^13^, ^14^, ^15^ As a result, VPA increases the LTG concentration and risk of rash^6^; therefore, when used in combination with VPA, the starting LTG dose should be significantly lower than the usual dose.^16^, ^17^

Previously, a variety of drugs have been reported as UGT inhibitors in vitro.^18^, ^19^ These include nonsteroidal anti‐inflammatory drugs, benzodiazepines, tricyclic antidepressants, and immunosuppressants. The interaction between LTG and VPA is widely known, whereas no other drugs have been proven to increase the serum concentrations of LTG in humans by inhibiting UGT. Bipolar disorder and epilepsy are often treated using a combination with other medications.^20^, ^21^ Furthermore, several drugs for other diseases may be used in combination, which may increase the risk of rashes in clinical practice. Therefore, drugs that affect rashes must be identified to ensure safe treatment. Accordingly, we used multiple sets of clinical data to identify concomitant drugs that increase the risk of LTG‐induced rashes. The aim of this study was to determine the impact of other drugs used in combination with LTG on the risk of rash.

## Methods

### Risk of rash associated with LTG use in combination with other drugs using VigiBase

#### Data sources

VigiBase is a World Health Organization global pharmacovigilance database designed to collect and analyze adverse drug reaction reports worldwide.^22^ This study uses the VigiBase data set collected in December 2021. Since VigiBase contains duplicate reports, those considered as duplicates by the Uppsala Monitoring Centre were excluded from the analysis using statistical algorithms. Informed consent was not required because no therapeutic intervention was conducted and an anonymized global open database was used.

SCAR was defined using the four terms of Severe Cutaneous Adverse Reactions (SMQ 20000020)” according to the Medical Dictionary for Regulatory Activities (MedDRA) version 26.0 (Table S1). The risk of adverse events was assessed using the reporting odds ratios (RORs) and 95% confidence intervals (CIs). Patients administered LTG were classified into four groups: those who (A) used a certain drug and reported SCAR, (B) used a certain drug and did not report SCAR, (C) did not use a certain drug and reported SCAR, and (D) did not use a certain drug and did not report SCAR. Based on the following equations, the RORs and 95% CIs were calculated for each drug.

ROR=AD/BC

$$95\%\mathrm{CI}=\exp\left(\ln\;\mathrm{ROR}\pm 1.96\surd 1/A+1/B+1/C+1/D\right)$$

A significant association between drug use and an increased incidence of SCAR was determined if the lower limit of the 95% CI exceeded 1. Conversely, a significant association between drug use and a decreased incidence of SCAR was determined if the upper limit of the 95% CI was <1.

### Retrospective case‐control study of LTG‐induced rashes

#### Design and population

We performed a retrospective case‐control study of patients who underwent LTG treatment at Tokushima University Hospital, Tokushima, Japan, or Asahikawa Medical University Hospital, Hokkaido, Japan, between 1 January 2009, and 31 December 2019, with the approval of the ethics committees of Tokushima University Hospital (reference number 3856) and Asahikawa Medical University Hospital (reference number 21070).

This study was conducted in accordance with the principles of the Declaration of Helsinki. As the present study was retrospective in nature and used existing information, an opt‐out option was displayed on the institution's website as a surrogate for informed consent for eligible patients, according to the Japanese Governmental guidelines.^23^ Patients were included if they initiated LTG treatment at one of the participating centers between 1 January 2009, and 31 December 2019, and were at least 18 years old. Patients were excluded if they were administered LTG for indications other than bipolar disorder or epilepsy and had never taken LTG after initiation.

#### Data collection and definitions

We collected baseline data related to sex, age, initiating dose of LTG, concomitant VPA use, concomitant enzyme inducer (medications that decrease the concentration of LTG; Table S2) use, other concomitant medications, and disease history at the initiation of LTG treatment obtained through electronic medical records from Tokushima University Hospital and Asahikawa Medical University Hospital. The presence or absence of rash with LTG was determined from each hospital's medical records, referring to the term “Skin and Subcutaneous Tissue Disorders” in MedDRA version 26.0. Patients were followed up for a period of 24 weeks after the initiation of LTG to allow for sufficient evaluation of rash incidence. Follow‐up was terminated if the patient discontinued LTG during this period.

#### Propensity score matching analysis

As patients in this retrospective case‐control study were not randomized to groups treated with or without flunitrazepam (FNZ), propensity score matching (PSM) was performed to decrease variability between cohorts.

Six factors (sex, age, disease, LTG starting dose, concomitant VPA use, and concomitant enzyme inducer use) were selected as important demographic characteristics associated with LTG‐induced rash in a logistic regression model used to calculate propensity scores (Table S3). A 1:1 PSM analysis was performed using the nearest neighbor matching method with a caliper of 0.20 to reduce the potential bias of patient characteristics. Patients who did not meet the matching criteria were excluded.

### Prospective observational study of LTG concentration

#### Population and data collection

We performed a prospective observational study in patients who underwent LTG treatment at Tokushima University Hospital, Tokushima, Japan, between 1 May 2023, and 30 April 2024, with the approval of the ethics committee of Tokushima University Hospital (reference number 4228). This study was conducted in accordance with the Declaration of Helsinki. The participants provided written informed consent before enrollment. Patients were included if they were taking LTG at the same dose for at least 2 weeks and were at least 18 years old. Patients were excluded if they took concomitant medications that affected the LTG concentration (VPA, enzyme inducers). Serum samples for trough values could not be obtained.

We collected data related to sex, age, disease history, weight, LTG dose, concomitant medications, and laboratory values using electronic medical records from the Tokushima University Hospital. Laboratory values included aspartate aminotransferase (AST), alanine aminotransferase (ALT), creatinine, and serum urea nitrogen (BUN). Data on smoking, adherence, and all concomitant medications were also obtained at the time of serum sampling.

The concentration‐to‐dose (C/D) ratio (μg/mL per mg/kg) was calculated by dividing the trough LTG concentration (μg/mL) by the LTG dose per kg body weight (mg/kg). The N2‐glucuronide/LTG ratio was calculated by dividing the trough LTG concentration (μg/mL) by the LTG N2‐glucuronide concentration (μg/mL).

#### Measurement of serum LTG concentration

Serum samples collected 9 to 16 h after the last oral dose of LTG were used to determine trough values.^24^ The serum concentrations of LTG and its metabolite, N2‐glucuronide, were quantified using liquid chromatography–tandem mass spectrometry (LC–MS/MS). The analytical procedure utilized an LC–MS/MS 8050 device (Shimadzu) with detection conducted in positive ion mode through electrospray ionization. Selective reaction monitoring was employed to detect LTG, N2‐glucuronide, and the internal standard substance lamotrigine‐13C3, with the precursor and product ions (m/z) set at 255.9 > 211.0, 431.9 > 256.1, and 258.9 > 214.2, respectively. The target compounds were separated by high‐performance liquid chromatography using a Kinetex C18 column (100 mm × 2.1 mm, 2.6 μm; Phenomenex) at a column temperature of 40°C and a flow rate of 0.2 mL/min. The mobile phase consisted of a solution of 0.1% formic acid in water and 0.1% formic acid in acetonitrile, delivered in gradient mode. Sample preparation involved solid‐phase extraction using a MonoSpin C18 column (GL Sciences). A 50‐μL aliquot of the serum sample was mixed with a 50‐μL solution of the internal standard (2 μg/mL in 2% formic acid in water) and 200 μL of 2% formic acid in water. Subsequently, the prepared mixture was applied to a MonoSpin C18 column that had been preconditioned with 2% formic acid in water, followed by acetonitrile. Subsequently, the column was washed with a solution of 2% formic acid in water and elution was performed using a mixture of 0.1% formic acid in water and 0.1% formic acid in acetonitrile (3:7 v/v). The resulting eluate was used as an analytical sample. The reference standards of LTG (>99.5%, LKT Laboratories), LTG N2‐glucuronide (>99%, Santa Cruz Biotechnology), and LTG‐13C3 (>95%, LGC Standards) were dissolved in methanol at a concentration of 1 mg/mL and subsequently diluted as required for utilization. A calibration curve was constructed with concentrations of 25, 10, 4, 1.6, 0.64, and 0.256 μg/mL.

### Statistical analysis

Differences between groups were analyzed using the parametric unpaired *t* test or nonparametric Mann–Whitney *U* test, as applicable. Categorical variables were compared using Fisher exact test or χ^2^ test. Statistical significance was set at <0.05. Statistical analysis of the data was performed using EZR version 4.2.2.^25^

The retrospective study was approved by the ethics committees of Tokushima University Hospital (reference number 3856) and Asahikawa Medical University Hospital (reference number 21070). The prospective study was approved by the ethics committee of Tokushima University Hospital (reference number 4228).

For the retrospective study, an opt‐out option was displayed on the institution's website as a surrogate for informed consent for eligible patients, according to the Japanese Governmental guidelines. For the prospective study, the participants provided written informed consent before enrollment.

## Results

### Medical big data analysis with VigiBase

Among the 27,994,584 spontaneous adverse event reports submitted, 108,962 cases involved LTG. Based on these reports, the ROR for SCARs was compared among cases reporting combination treatment with drugs that inhibit UGT (Table S4).^18^, ^19^

In total, 1.93% of patients who underwent LTG reported SCARs. We excluded combinations of LTG with other drugs, with <100 reported cases to ensure the reliability of the analysis. The RORs for drugs that inhibited UGT are presented in Table 1. The RORs of the combinations with VPA, FNZ, and nitrazepam were 1.48 (95% CI, 1.18–1.85), 8.12 (95% CI, 6.05–10.92), and 2.98 (95% CI, 1.80–4.94), respectively, all significantly increasing the ROR for LTG‐related SCAR.

**Table 1:** Effect of risk drug candidates on the occurrence of lamotrigine‐induced SCAR using the VigiBase analysis

Drug	SCAR without the drug (%)	SCAR with the drug (%)	ROR (95% CI)
Valproic acid	1.91 (2024/106062)	2.79 (81/2900)	1.48 (1.18–1.85)
Flunitrazepam	1.89 (2053/108578)	13.54 (52/384)	8.13 (6.05–10.92)
Nitrazepam	1.92 (2089/108672)	5.52 (16/290)	2.98 (1.80–4.94)
Promethazine	1.93 (2091/108281)	2.06 (14/681)	1.07 (0.63–1.81)
Ciclosporin	1.93 (2099/108605)	1.68 (6/357)	0.87 (0.39–1.95)
Indometacin	1.93 (2103/108823)	1.44 (2/139)	0.74 (0.18–2.99)
Diclofenac	1.94 (2098/108393)	1.23 (7/569)	0.63 (0.30–1.33)
Tacrolimus	1.93 (2103/108799)	1.23 (2/163)	0.63 (0.16–2.54)
Clonazepam	2.00 (1988/99500)	1.24 (117/9462)	0.61 (0.51–0.74)
Ibuprofen	1.95 (2078/106446)	1.07 (27/2516)	0.54 (0.37–0.80)
Lorazepam	1.97 (2056/104387)	1.07 (49/4575)	0.54 (0.41–0.72)
Naproxen	1.94 (2097/108096)	0.92 (8/866)	0.47 (0.23–0.95)
Diazepam	1.97 (2082/105881)	0.75 (23/3081)	0.37 (0.25–0.57)
Fluconazole	1.94 (2102/108513)	0.67 (3/449)	0.34 (0.11–1.06)
Ranitidine	1.94 (2101/108225)	0.54 (4/737)	0.28 (0.10–0.74)
Propranolol	1.95 (2099/107856)	0.54 (6/1106)	0.27 (0.12–0.61)
Furosemide	1.96 (2094/106838)	0.52 (11/2124)	0.26 (0.14–0.47)
Amitriptyline	1.95 (2103/108095)	0.23 (2/867)	0.12 (0.03–0.47)

*Note*: Drugs with <100 adverse events reported in combination with lamotrigine are excluded.

Abbreviations: CI, confidence Interval; ROR, reporting odds ratio; SCAR, severe cutaneous adverse reaction.

### Patient characteristics in the retrospective case‐control study

VigiBase results showed that FNZ increased the ROR of LTG‐induced skin damage. To validate this result with clinical data, a retrospective study and a prospective observational study were conducted.

**Fig. 1:**
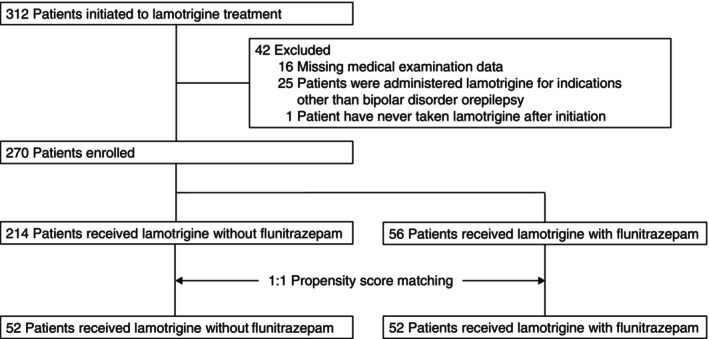
Flow chart of patient selection in the retrospective case‐control study.

A total of 312 patients underwent LTG treatment between 1 January 2009, and 31 December 2019. Of these, 42 patients were excluded because they had missing medical examination data, were administered LTG for indications other than bipolar disorder or epilepsy, or had never received LTG after treatment initiation. A total of 270 patients who met the inclusion criteria were enrolled in this study. The mean ± standard deviation of the follow‐up period was 17.4 ± 8.90 weeks. There were 214 patients in the LTG without FNZ group and 56 patients in the LTG with FNZ group (Fig. 1). In the LTG with FNZ group, 87.5% (49 of 56) of the patients took FNZ routinely before bedtime, and 12.5% (seven of 56) took FNZ as needed. A rash occurred in 58 of the 270 enrolled patients. Most rashes were mild, but one patient with a severe case required admission to the intensive care unit and required steroid treatment. There were significant differences in the incidence of rashes (LTG without FNZ group, 18.2%; LTG with FNZ group, 33.9%), age, and the diagnosis between groups (Table 2).

**Table 2:** Patient characteristics of the overall series and propensity score–matched pairs in the retrospective case‐control study

	Before propensity score matching				After propensity score matching			
Variable	LTG without FNZ (*n* = 214)	LTG with FNZ (*n* = 56)	*P‐*value	SMD	LTG without FNZ (*n* = 52)	LTG with FNZ (*n* = 52)	*P‐*value	SMD
Rash (%)	39 (18.2)	19 (33.9)	0.017^†^	0.364	8 (15.4)	19 (36.5)	0.024^†^	0.497
Sex (male/female)	70/144	19/37	0.874^†^	0.026	19/33	17/35	0.837^†^	0.081
Age (years)	39.0 [28.3–55.8]	52.5 [37.0–62.3]	0.002^‡^	0.444	44.5 [33.0–64.8]	50.0 [36.5–63.0]	0.654^‡^	0.048
Diagnosis (%) Epilepsy	136 (63.6)	8 (14.3)	<0.001^†^	1.171	8 (15.4)	8 (15.4)	1.000^†^	<0.001
Bipolar disorder	78 (36.4)	48 (85.7)			44 (84.6)	44 (84.6)		
Starting dose ≦12.5 mg/d (%)	90 (42.1)	22 (39.3)	0.762^†^	0.056	18 (34.6)	20 (38.5)	0.839^†^	0.080
VPA in combination (%)	63 (29.4)	11 (19.6)	0.179^†^	0.229	9 (17.3)	10 (19.2)	1.000^†^	0.050
Enzyme inducers in combination (%)	59 (27.6)	10 (17.9)	0.169^†^	0.233	7 (13.5)	6 (11.5)	1.000^†^	0.058

*Note*: Values are expressed as median [interquartile range] or number of patients (%).

Abbreviations: FNZ, flunitrazepam; LTG, lamotrigine; SMD, standardized mean difference; VPA, valproic acid.

^†^ Significance was determined using Fisher exact test.

^‡^ Significance was determined using Mann–Whitney *U* test.

### Propensity score–adjusted results

The area under the curve for the propensity score in patients who received LTG with FNZ with six covariates was 0.792. We obtained 52 pairs by matching propensity scores using a caliper value of 0.2. Significant differences in age and disease‐related factors that affected rash development observed before matching were eliminated from the pairs after matching. After the 1:1 PSM analysis, the presence of rashes was significantly higher in the LTG with FNZ group (19 of 52, 36.5%) than in the LTG without FNZ group (eight of 52, 15.4%) in the 52 matched pairs. No significant differences in other patient characteristics were observed among the 52 matched pairs. All covariates were balanced after PSM, with a standardized mean difference (SMD) <0.1 (Table 2). PSM analysis revealed an increased risk of rash in patients concomitantly treated with FNZ.

### Patient characteristics in the prospective observational study

Seventeen patients who underwent LTG treatment with the same dose for at least 2 weeks participated in the study. Of these, one patient was excluded because serum samples could not be obtained to determine trough values. Sixteen patients who received LTG for the treatment of bipolar disorder were included in the analysis: nine in the LTG without FNZ group and seven in the LTG with FNZ group. In the LTG with FNZ group, all patients took FNZ routinely before bedtime. The patient characteristics are presented in Table 3. No patients had abnormal laboratory values (AST, ALT, creatinine, and BUN). The median LTG dose was not significantly different between the LTG without FNZ group (200 mg/d; interquartile range [IQR], 100–300 mg/d) and LTG with FNZ group (200 mg/d; IQR, 150–300 mg/d). There were no significant differences in patient characteristics between groups.

**Table 3:** Patient characteristics in the prospective observational study

Variable	LTG without FNZ (*n* = 9)	LTG with FNZ (*n* = 7)	*P*‐value
Sex (male/female)	5/4	5/2	0.633^†^
Age (years)	48.0 [42.0–49.0]	49 [45.5–59.5]	0.396^‡^
Body weight (kg)	76.6 [61.8–83.1]	77.7 [70.9–84.8]	0.837^‡^
Height (cm)	166.0 [157.8–169.5]	167.7 [162.1–173.3]	0.758^‡^
AST (IU/L)	23.0 [17.0–28.0]	22 [20.5–30.5]	0.710^‡^
ALT (IU/L)	20.0 [17.0–42.0]	31.0 [22.5–42.5]	0.751^‡^
Serum creatinine (mg/dL)	0.88 [0.69–1.02]	0.84 [0.78–0.85]	0.426^‡^
BUN (mg/dL)	12 [11.0–13.0]	14 [12.0–14.5]	0.630^‡^
LTG dose (mg/d)	200 [100–300]	200 [150–300]	1.000^‡^
Smoking	2 (22.2)	1 (14.3)	1.000^†^
Concomitant medications	6 [3.0–7.0]	6 [4.5–8.5]	0.262^‡^

*Note*: Values are expressed as median [interquartile range] or number of patients (%).

Abbreviations: ALT, alanine aminotransferase; AST, aspartate aminotransferase; BUN, serum urea nitrogen; FNZ, flunitrazepam; LTG, lamotrigine.

^†^ Significance was determined using Fisher exact test.

^‡^ Significance was determined using Mann–Whitney *U* test.

### Prospective observational study results

**Fig. 2:**
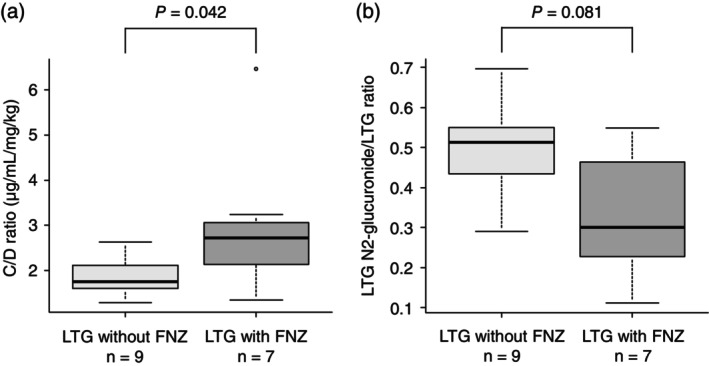
Box and whisker plot of the C/D ratio and N2‐glucuronide/LTG ratio for the LTG without FNZ group and the LTG with FNZ group in the prospective observational study. (a) Box and whisker plot of the C/D ratio for the LTG without FNZ group vs the LTG with FNZ group. (b) Box and whisker plot of the N2‐glucuronide/LTG ratio for the LTG without FNZ group vs the LTG with FNZ group. C/D, concentration‐to‐dose (μg/mL per mg/kg); FNZ, flunitrazepam; LTG, lamotrigine. Significance was determined using Mann–Whitney *U* test.

The median of C/D ratio was 2.60 μg/mL per mg/kg (IQR, 1.94–3.02 μg/mL per mg/kg) and 1.67 μg/mL per mg/kg (IQR, 1.44–1.91 μg/mL per mg/kg) in patients treated LTG with and without FNZ, respectively. The C/D ratio was significantly higher in the LTG with FNZ group than in LTG without FNZ group (*P* < 0.05; Fig. 2a).

The median of The N2‐glucuronide/LTG ratio was 0.30 (IQR, 0.23–0.46) and 0.51 (IQR, 0.43–0.55) in patients treated with LTG with and without FNZ. The N2‐glucuronide/LTG ratio showed a trend toward a significant decrease in the LTG with FNZ group compared with the LTG without FNZ group, with a *P‐*value of 0.081, indicating a near‐significant difference (Fig. 2b).

The FNZ dose in the LTG with FNZ group was within the indication for medical treatment, with a median of 1.00 mg/d (IQR, 1.00–2.00 mg/d). Furthermore, the FNZ serum concentration was 3.28 ng/mL (2.20–4.84 ng/mL). The serum concentrations of each drug are listed in Table S5.

## Discussion

This study utilized multiple sets of clinical data to identify the concomitant medications that increased the risk of LTG‐induced rashes. The results of the VigiBase analysis and retrospective case‐control study showed that concomitant FNZ use increased the incidence of LTG‐induced rashes. In addition, the prospective observational study showed that concomitant FNZ use increased LTG concentration. These results suggest that FNZ may inhibit UGT, leading to increased LTG concentration and a heightened risk of LTG‐induced rash. Notably, previous studies have reported no significant increase in LTG concentrations by drugs other than VPA. However, the present study demonstrates that FNZ significantly elevates the C/D ratio of LTG. Given that FNZ is commonly used as a sleep aid, its potential risk of exacerbating LTG‐induced skin reactions should be carefully considered in clinical practice.

LTG is widely used in the fields of psychiatry and neurology. LTG‐induced rashes may develop into fatal SCARs, such as Stevens–Johnson syndrome and toxic epidermal necrolysis. For LTG to be used safely, the risk of rashes must be minimized. The mechanism of LTG‐induced rashes is not fully understood but has been reported to involve sulfotransferase pathways, LTG itself, drug‐specific T cells, and the human leukocyte antigen.^26^, ^27^, ^28^, ^29^ A rapid increase in LTG concentration during the early stages of administration is associated with the development of rashes.^6^, ^8^, ^9^, ^10^ Since concomitant VPA use increases LTG concentration, the LTG dose is controlled to prevent the onset of rash. The treatment of bipolar disorder and epilepsy is often combined with other medications, which may affect the risk of rash.

In the medical big data analysis using VigiBase, concomitant FNZ and VPA use was shown to significantly increase the ROR of SCAR. Among other benzodiazepines, concomitant nitrazepam use also increased the ROR of SCAR, whereas diazepam, clonazepam, and lorazepam did not. The differences in RORs among benzodiazepines may be caused by the varying degrees of UGT inhibition and UGT enzyme families.^30^, ^31^ However, no in vitro studies directly comparing effects of UGT inhibition among benzodiazepines and examining the effect of FNZ on LTG metabolism have been reported.

This retrospective case‐control study showed an increased incidence of LTG‐induced rash with concomitant FNZ use in an analysis that considered the factors affecting rash. The incidence of rash reported by Watanabe *et al*.^32^ was 13.7% (61 of 445) in patients with bipolar disorder taking LTG, whereas that in patients taking LTG without FNZ in our PSM analysis was 15.4% (eight of 52). The incidence of LTG‐induced rash was similar to that reported in previous studies and was considered adequately evaluated. When LTG was combined with FNZ, the incidence of rashes significantly increased in the PSM analysis, suggesting an additional effect of FNZ on rash risk.

To the best of our knowledge, this is the first prospective observational study to report that medications other than VPA increase LTG concentration in humans. LTG exhibits first‐order linear pharmacokinetics, with its concentration increasing in a dose‐dependent manner.^33^ Drug interactions have been reported to alter C/D ratio, with the C/D ratio increasing approximately 2.2‐fold when LTG and VPA were combined.^34^ The C/D ratio of the LTG without FNZ group in our study and of the LTG monotherapy group in a controlled clinical trial in Japanese patients showed similar results.^34^ In our study, we found that FNZ significantly increased the LTG C/D ratio. We also examined the effects of LTG on its metabolites. The N2‐glucuronide/LTG ratio reflects LTG glucuronide conjugation and decreases as the metabolism slows down. This ratio is useful for identifying the metabolic effects of concomitant medications and pregnancy.^35^, ^36^ The N2‐glucuronide/LTG ratio of 0.54 in the LTG group found by Yang *et al*.^36^ was similar to the respective value of the LTG without FNZ group in our study (0.51). The N2‐glucuronide/LTG ratio was lower in the LTG with FNZ group than in the LTG without FNZ group. These results suggest that FNZ inhibits LTG metabolism.

LTG is primarily metabolized by UGT1A4 and various UGT isoforms, such as UGT1A3, UGT2B7, and UGT2B10, are also involved in its metabolism.^31^, ^37^, ^38^ The mechanism by which VPA increases LTG concentration has been reported to involve the inhibition of UGT2B7 and 2B10.^13^, ^38^ Some reports have indicated that FNZ inhibits UGT. For example, it was shown that FNZ exhibits potent competitive inhibition of rat liver morphine UGT activity.^39^ Another study showed that FNZ inhibited glucuronidation of 3′‐azido‐3′‐deoxythymidine in the human liver.^40^ In addition, FNZ inhibited UGT2B7 as well as VPA in vitro.^41^ In our prospective observational study, FNZ increased the LTG concentration, likely in association with UGT inhibition.

Our study has some limitations. In the VigiBase analysis, the ROR of SCAR was significantly increased when nitrazepam was combined with LTG. We attempted to investigate the risk of rash with concomitant nitrazepam use, but both retrospective and prospective studies were unable to evaluate the small number of patients receiving nitrazepam concomitantly. The effects of benzodiazepines other than FNZ also need to be tested in large cohort studies.

A retrospective cohort study reported that rashes during LTG administration were not always related to drug hypersensitivity.^42^ Other rashes unlikely to be caused by LTG were more common in children than adults.^42^ Our retrospective case‐control study did not include children, and the occurrence of other rashes unlikely to be caused by LTG is considered low.

In this prospective study, we evaluated factors affecting LTG concentration but were unable to examine UGT gene polymorphisms. UGT1A4 and UGT2B7 genetic polymorphisms may affect LTG concentration.^43^, ^44^ Moreover, different frequencies of the UGT mutant alleles have been reported among different ethnic groups.^45^ Furthermore, a higher risk of SCAR owing to LTG has been reported in Asian patients.^46^ Another limitation of our study is that it only included Japanese patients. Validation in a larger multiethnic sample is needed. In addition, since the LTG maintenance dose was evaluated at the trough LTG steady‐state concentration, it was not possible to prospectively examine the effect of LTG concentration during the early administration period on the risk of rash. Further detailed prospective studies are required.

## Conclusions

This study showed that FNZ increased LTG serum concentration and increased the risk of rash. Concomitant use of FNZ should be carefully considered in patients using LTG. This is the first report to clearly demonstrate an interaction with LTG in a drug other than VPA. Given that FNZ is a potent benzodiazepine used as a sleep medication, these results have implications for the choice of sleep medication in patients receiving LTG. When VPA is used in combination with LTG, the risk of rash is controlled by careful titration, and special attention must be paid to the incidence of rash in clinical practice. Furthermore, when LTG is used in combination with FNZ, LTG dosing should be carefully adjusted to prevent rash.

## Disclosure statement

The authors declare no conflicts of interest associated with this study.

## Author contributions

Study conception and design: H.B., M.G. Performance of the experiments and data acquisition: H.B., M.G., M.N., K.H., Y.N., T.Y., C.M., K.M. Analysis and interpretation of data: H.B., M.G., M.N., K.H., K.K. (Kei Kawada), T.Y., M.K., A.O., K.T., K.K. (Kohei Kitagawa), T.N., F.A., K.Y., M.C., S.N. Drafting the work or critically revising it for relevant intellectual content: H.B., M.G. K.H., Y.I., T.N., Y.T., S.N., K.I.

## Supporting information


**Data S1.** Supporting Information.

## Data Availability

All data are available in the main text or the supplementary materials.
