# Supplementary material for: Flunitrazepam increases the risk of lamotrigine‐induced cutaneous adverse reactions: Combined analysis of medical big data and clinical research
Source: Psychiatry Clin Neurosci. 2025 Jul 17;79(10):629–35. doi: 10.1111/pcn.13866 (PMC12498123; doi:10.1111/pcn.13866)
Supplement: Supplementary file 1 — Data S1. Supporting Information. [file PCN-79-629-s001.docx]

Supporting information

Table S1. Search terms for severe cutaneous adverse reactions event in the analysis of VigiBase.

| preferred term |
| --- |
| Oculomucocutaneous syndrome |
| SJS-TEN overlap |
| Stevens-Johnson syndrome |
| Toxic epidermal necrolysis |

Table S2. Enzyme inducers have been clinically proven to decrease lamotrigine concentration.

| Phenytoin |
| --- |
| Carbamazepine |
| Phenobarbital |
| Primidone |
| Rifampicin |
| Lopinavir/ritonavir |
| Atazanavir/ritonavir |
| Ethinyloestradiol/levonogestrel combination (Hormonal Contraceptives) |

Table S3. Derivation of propensity score (combined with flunitrazepam) equations from covariates using logistic regression.

| covariates | coefficient | 95% CI | p value |
| --- | --- | --- | --- |
| Sex (female) | 0.03449 | 0.515-2.080 | 0.923 |
| Age | 0.01859 | 0.999-1.040 | 0.064 |
| Diagnosis (bipolar disorder) | 2.81725 | 5.710-49.000 | <0.01 |
| Starting dose ≦12.5 mg/day | -0.39362 | 0.298-1.530 | 0.344 |
| VPA in combination | -0.72972 | 0.181-1.280 | 0.144 |
| Enzyme inducers in combination | 1.03946 | 0.921-8.680 | 0.069 |

Abbreviations: CI, Confidence Interval.

Table S4. List of drugs that inhibit UDP-glucuronosyltransferase.

| Valproic Acid |
| --- |
| Diazepam |
| Lorazepam |
| Flunitrazepam |
| Clonazepam |
| Nitrazepam |
| Nortriptyline |
| Clomipramine |
| Amitriptyline |
| Diclofenac |
| Flurbiprofen |
| Naproxen |
| Indometacin |
| Acetaminophen |
| Mefenamic Acid |
| Flufenamic Acid |
| Ibuprofen |
| Ketoprofen |
| Tacrolimus |
| Ciclosporin |
| Ethinyl Estradiol |
| Testosterone |
| Probenecid |
| Chloramphenicol |
| Naloxone |
| Morphine |
| Methadone |
| Furosemide |
| Diaphenylsulfone |
| Cimetidine |
| Ranitidine |
| Fluconazole |
| Atovaquone |
| Propranolol |
| Promethazine |

Table S5. The blood concentrations of each drug in the prospective observational study.

| Variable | LTG without FNZ  (n = 9) | LTG with FNZ  (n = 7) | p value |
| --- | --- | --- | --- |
| LTG concentration (µg/mL) | 5.10 [3.40-5.40] | 6.80 [5.50-7.15] | 0.071 |
| LTG N2-glucuronide (µg/mL) | 2.62 [1.70-3.34] | 2.02 [1.46-3.34] | 0.758 |
| FNZ dose (mg/day) | 0 | 1.00 [1.00-2.00] | NA |
| FNZ concentration (ng/mL) | 0 | 3.28 [2.20-4.84] | NA |

Note: median [interquartile range]

Abbreviations: FNZ, flunitrazepam; LTG, lamotrigine; NA, not applicable.

Significance is determined using Mann-Whitney U test.
